# Self-Monitoring Physical Activity, Diet, and Weight Among Adults Who Are Legally Blind: Exploratory Investigation

**DOI:** 10.2196/42923

**Published:** 2022-12-12

**Authors:** Kamilla Miller, Gerald J Jerome

**Affiliations:** 1 Department of Kinesiology Towson University Towson, MD United States

**Keywords:** blindness, visually impaired, obesity, weight loss, weight management, physical activity, digital health intervention, telehealth, health support, mobile phone

## Abstract

**Background:**

Obesity is a global pandemic. Lifestyle approaches have been shown effective for weight loss and weight loss maintenance. Central to these evidence-based approaches are increased physical activity, decreased caloric intake, regular self-weighing, and the tracking of these behaviors.

**Objective:**

This exploratory descriptive study surveyed adults who are legally blind to identify strategies related to tracking physical activity, diet, and weight. These health behaviors are essential components to evidence-based weight loss programs. We also identified areas where we can better support adults who are legally blind in their independent efforts to change these behaviors and improve their health.

**Methods:**

Participants (≥18 years of age) who self-identified as being legally blind were recruited using email announcements in low vision advocacy groups. They completed an interviewer-administered survey on the telephone and an in-person visit for standardized assessment of height and weight.

**Results:**

The participants (N=18) had an average age of 31.2 (SD 13.4) years; 50% (9/18) had normal weight (BMI 18.5 to <25); 44% (8/18) were female; 44% (8/18) were Black; and 39% (7/18) were Non-Hispanic White. Most participants (16/18, 89%) used their smartphone to access the internet daily, and 67% (12/18) had at least 150 mins of exercise per week. Although 78% (14/18) of the participants indicated tracking their weight, only 61% (11/18) could indicate how they tracked their weight, and 22% (4/18) indicated they tracked it mentally. Providing individuals with a talking scale was the most consistent recommendation (12/18, 67%) to facilitate independence in managing weight through lifestyle changes. Even though 50% (9/18) of the participants indicated using an app or electronic notes to track some portion of their diet, participants reported challenges with determining portion size and corresponding calorie counts. Most participants (17/18, 94%) reported using apps, electronic notes, smartphones, or wearable devices to track their physical activity. Although strategies such as using wearables and smartphones could provide measurements (eg, step counts) as well as recording data, they also pose financial and technology literacy barriers.

**Conclusions:**

Technology-based solutions were identified for tracking weight, diet, and physical activity for weight management. These strategies have financial and technology literacy barriers. A range of strategies for adopting and tracking health behaviors will be needed to assist individuals with varying skills and life experiences.

## Introduction

Obesity is a significant public health concern [1,2]. Adults with low vision are 1.5 times more likely to be obese compared to the general population [3,4]. Although there is robust data on the effectiveness of a lifestyle approch to weight management [5], those with vision impairements may have been excluded from weight loss trials due to inaccessible enrollment processes or study materials. The extent to which adults with low vision have been excluded is likely in proportion to their degree of vision impairment and the level of accommodation that is needed. Some individuals with low vision may need basic accommodations, such as larger print or the use of a magnifier. Those with greater vision impairment, who use a screen reader, would need accessible documents and web-based forms. Accessibility has been defined as providing equivalent user experience for those with a disability [6]. In this case, accessible documents refer to documents formatted to provide an equivalent experience when reading or using a screen reader. Providing an accessible enrollment experience would require the development of enrollment processes and study materials to ensure documents and web-based interfaces are accessible. Although studies often indicate their materials were culturally appropriate, it is less clear if the materials were accessible to those with a disability, especially those who are legally blind. In addition, few, if any, weight loss studies have focused on adults with low vision or provided this group with tailored approaches to help them monitor, track, and problem solve challenges related to health behavior change for weight loss [7]. As such, there has been scant attention paid to tailoring strategies to help adults with low vision achieve and maintain a healthy weight.

Lifestyle approaches to weight management include recommendations of increased exercise, caloric restriction, self-weighing, and tracking these health behaviors [8]. Self-monitoring, or being aware of these health behaviors, is important for lifestyle-based weight management. Tracking, or recording data on these behaviors, provides individuals with additional support and opportunities. Tracking these behaviors allows for an objective evaluation of progress, which can provide encouragement. By examining the data provided from tracking, an individual can develop an understanding of the connection between these behaviors and weight change. This can inform problem-solving and assist with increased adoption of and adherence to these health behaviors.

A simple approach to tracking is to keep a record (eg, time spent exercising) on a piece of paper. Individuals who are blind could use braille notes or simple technology such as voice memos. Additional technology-based approaches include using smartphone apps to monitor and track health behaviors. For example, wearables (eg, smart watches) provide frictionless data capture to estimate physical activity, sedentary time, and sleep time. A large number of smartphone apps and technologies are designed to help individuals adopt and adhere to weight loss–related behaviors [9-11]. Unfortunately, weight loss apps do not always provide support across the full complement of evidence-based recomendations [12,13]. Moreover, the accessibility of health apps has been shown to be limited [14]. Despite a proliferation of health-related apps, it is not clear that adults who are legally blind have access to evidence-based support for weight loss.

In this exploratory descriptive study, we survey adults who are legally blind to identify strategies related to tracking physical activity, diet, and weight. We also identify areas where we can better support these adults in their independent efforts to change these behaviors and improve their health.

## Methods

### Recruitment

We recruited participants through email announcements for individuals who were legally blind and willing to answer questions about their diet and exercise regardless of their interest in weight management. Email announcements were posted on campus and among local low vision advocacy groups. Inclusion criteria included being ≥18 years of age, legally blind, willing to complete the surveys, and able to meet in person for height and weight assessment. Exclusion criteria included being deaf and blind or having a medical condition where caloric restriction or physical activity may be contraindicated, including but not limited to recent cardiac event, chemotherapy, and end stage renal disease. Participants who completed the telephone survey and the in-person visit for height and weight assessment were eligible to receive a US $30 gift card.

### Ethics Approval

All procedures were approved by the Towson University Institutional Review Board (1807037548), and the study was conducted in accordance with the Helsinki Declaration. All participants provided informed consent.

### Measures

Surveys were interviewer-administered over the telephone. Participants self-identified as legally blind. The telephone survey included items from established questionnaires. Questions to assess demographics, fruit and vegetable consumption, and technology use were from the National Cancer Institute, Health Information National Trends Survey [15]. The Godin Leisure Time Physical Activity Questionnaire was used to assess physical activity levels [16,17]. After these close-ended questionnaire items, participants were asked open-ended questions specific to this study. Study staff provided context for the open-ended questions. They explained the following:

Studies have shown that tracking a few key behaviors is an important element in safe, effective, long-term weight management.

A definition of tracking was provided, as follows:

[tracking is] recording a number, for example weight in pounds or minutes of activity in a day, such that you could review that number later.

Participants were then asked a series of three questions regarding past tracking, successes and barriers, and suggestions for assistance. The questions started with “Have you ever tracked your weight, and if so, how did you do it?” Participants were asked to describe any methods or techniques they had used in the past to track their weight. Next, participants were asked to share any successes or barriers they experienced when tracking their weight. The last question in the sequence was the following:

If we were developing a weight management program for adults with low vision and wanted to encourage people to track their weight, what is the most important thing we could do to help?

The sequence of three questions was repeated two more times, once focused on tracking diet or calories and once focused on tracking physical activity. After completion of the surveys, participants had a single in-person visit, where standardized procedures were used to assess height and weight in light street clothes without shoes.

### Analytic Plan

Participant characteristics were summarized using descriptive statistics, including percentages, means, and standard deviations. Minutes of moderate to vigorous physical activity (MVPA) were calculated, and data were reported on the number of participants who met the national guidelines of at least 150 mins per week of MVPA [18]. An inductive coding approach was used to examine the open-ended questions [19]. For each item, an initial review of the responses was used to develop themes. A subsequent review of the items was used to determine the prevalence of the themes among the respondents. Any discrepancy in coding was resolved by discussion among the coders. Results included the prevalence of the themed items presented as a percentage of the total sample size.

## Results

### Participants

The participants (N=18) had an average age of 31.2 (SD 13.4) years; 44% (8/18) were female; 44% (8/18) were Black; 39% (7/18) were Non-Hispanic White; and 17% (3/18) were classified as “other race/ethnicity” to maintain confidentiality. Based on BMI classifications, 50% (9/18) had normal weight (BMI 18.5 to <25); 22% (4/18) were overweight (BMI 25 to <30); and 28% (5/18) were obese (BMI ≥30). Participants reported the consumption of fruits and vegetables as 1.0 (SD 0.8) and 1.4 (SD 1.0) servings per day, respectively, and 67% (12/18) had at least 150 mins per week of MVPA. Most participants (16/18, 89%) reported accessing the internet daily through a smartphone; the other 2 (11%) participants reported accessing the internet daily at work and occasionally through their smartphone. Most participants (14/18, 78%) reported use of a smartphone or tablet to track progress of a health-related goal.

### Tracking, Barriers, and Related Suggestions

More than half of the participants (14/18, 78%) reported tracking their weight; however, only 61% (11/18) could indicate specifically how they tracked it, with 11% (2/18) indicating an electronic note, 22% (4/18) indicating a smartphone or an app, and 22% (4/18) indicating “mentally.” One participant indicated their weight was tracked at the doctors. When asked about successes and barriers, the only barrier noted was not having an accessible scale, as identified by 33% (6/18) of the participants. When asked what would be most helpful to adults with low vision who wanted to track their weight, 67% (12/18) indicated an accessible scale, and 22% (4/18) indicated information and education. Two participants indicated they were not sure of the best way to track their weight to easily review changes across time.

More than half of the participants (14/18, 78%) reported tracking their diet, with 17% (3/18) using some type of electronic note, 33% (6/18) using a smartphone or an app, and 28% (5/18) tracking “mentally.” When asked about successes and barriers to tracking calories, 28% (5/18) indicated difficulty with seeing calorie counts, another 17% (3/18) indicated difficulty measuring, and another 22% (4/18) indicated that it was simply hard. Two participants listed meal prepping as a success but did not track calories related to these meals. When asked what would be helpful for adults with low vision who want to track dietary information (eg, calories), 33% (6/18) indicated accessible technology to determine nutritional information, and 56% (10/18) indicated basic information, education, and instruction to learn about nutrition and calories.

Most participants (17/18, 94%) reported tracking their physical activity, with 44% (8/18) using a smartphone or an app, 22% (4/18) using a wearable device, 22% (4/18) tracking “mentally,” and 1 (6%) using electronic notes. When asked about successes and barriers to tracking physical activity, those using wearable devices also listed the devices as successes. Among the barriers noted were not having enough time (5/18, 28%), cost (3/18, 17%), lack of a workout partner (3/18, 17%), and not having a place or blind friendly place to work out (3/18, 17%). When asked about how best to help people track their physical activity, 50% (9/18) recommended providing apps or wearables; 33% (6/18) suggested providing more fundamental support, such as information on how to exercise; and 56% (10/18) suggested providing a coach or exercise partner.

## Discussion

### Principal Findings

Although 78% (14/18) of participants indicated they tracked their weight, less than half used a method where they recorded a number that could be referenced later for personal use. For example, 22% (4/18) reported tracking their weight mentally. The strategy of using an app or electronic notes, which were the most common methods of tracking, has a financial barrier and requires technology literacy. Although the participants reported viable solutions for tracking weight, careful consideration is needed for the wide scale adoption of these options. An additional consideration is the need for a talking scale in order to obtain a weight. Participants indicated tracking of diet or caloric information was possible yet challenging. Participants were tracking aspects of diet using smartphones or apps as well as using electronic notes. However, gaining information about serving size and related calorie information was reported as a barrier. Participants were able to track physical activity through smartphones and apps. This has the advantage of frictionless data capture such as the phone automatically counting steps. However, this technology-based approach has the same financial and technology literacy barriers as noted previously. Overall, the identified technology-based solutions have limitations for adoption but appear aligned with evidence-based strategies of tracking weight, diet, and physical activity for weight management [8]. It should be noted there is significant heterogeneity among those who are legally blind. Some individuals may have lost their eyesight after establishing certain habits or memory of specific visual references. Others who were blind since birth may not have those visual references but could have a lifetime of skills related to independent travel, using braille, and using screen readers. As such, a range of strategies will be needed to assist individuals with varying skills and life experiences.

### Applications in Health Promotion

The most frequent and enthusiastic suggestion from participants was to provide talking scales. Over half of the participants indicated this was an important support component to a weight loss program for adults with low vision. This simple tool can raise daily awareness and empower independence in regular weight monitoring. This appears to be one of the most practical, fundamental, and easily addressed strategies. Providing scales to study participants is common in weight loss trials [20], and providing accessible scales should become standard to ensure all participants have equivalent support in weight loss programs. Over 20% of the participants indicated they mentally tracked weight, exercise, or diet. However, tracking should occur with documentation (eg, using electronic notes or apps) to avoid recall bias and allow for accurate review of data. It also allows data to be shared with a health care provider, wellness coach, or support group.

Although many consider health apps as a scalable approach for weight management, these apps may not provide a comprehensive approach to lifestyle-based weight loss [12,13]. There are additional problems with considering this approach for those who are legally blind. One problem is that some features, such as weight graphs, may not be accessible to those with vision loss [14]. Moreover, there are reported problems using voice control with popular health apps [14]. As noted earlier, the method of tracking should facilitate the examination of trends over time and allow for sharing of data with a wellness coach. Weight graphs are highly effective tools that quickly convey trends across time but do not assist those who are blind. Determining equivalent ways to share weight data is a challenging issue. In fact, developing equivalent data visualization experiences for those with vision impairments is a broad-based concern that extends beyond the current application [21]. Greater attention is needed to develop weight loss apps and programs that provide a full complement of evidence-based features that can be accessed by those with low vision. It is important that those with a disability, in this case individuals who are legally blind, have equivalent experiences with wellness programs compared to those without a disability.

Accessibility of nutrition information appeared to be a significant barrier to tracking diet. In addition, participants wanted help with identifying and preparing healthy foods that are aligned with specific diet goals as well as reading nutrition labels and selecting healthy foods in the supermarket. This is not directly related to tracking but is a barrier that needs to be addressed when promoting healthy weight among those with vision impairments. Problem-solving efforts for these barriers require additional steps based on an individual’s visual acuity and technological savvy. One participant wanted to gain weight and reported challenges reading labels for both nutrition and calories. It is not clear how many individuals with low vision may have the goal of gaining weight, but it deserves further consideration. This could be relevant to older adults who are the fastest growing demographic with respect to low vision [22] and have health concerns related to inadequate nutrition [23].

Some participants reported financial barriers associated with going to the gym, although it was not clear if the burden was associated with transportation or gym membership. Participants also indicated the need for basic information, workout partners, and accessible instruction on different exercise options. This was aligned with previous reports of barriers to physical activity for adults who are blind [24]. One participant indicated they did not always feel that fitness facilities were blind-friendly. The latter deserves further exploration because the Americans with Disabilities Act clearly addresses elements of a facility’s physical layout, but it does not address cultural changes to support diversity among gym members [25]. Creating a more inclusive environment may require partnering with fitness facilities in educating fitness staff [26].

### Strengths and Limitations

The modest sample size is a limitation of the study but is appropriate for an exploratory study. Some of the participants were technologically savvy, physically active, and conscientious of eating fruits and vegetables. This is both a strength and a limitation. The technologically savvy, health-conscious component of the sample is a strength, as these individuals were able to identify successful strategies, often technology-based, that they used in adopting healthy behaviors. These technologically savvy, health-conscious individuals are also a limitation of the study, as the percentage of these individuals in the current convenience sample are not necessarily representative of adults who are legally blind. Indeed, the percentages reported in this paper should not be considered representative of the population, and they have likely underestimated the amount of technology assistance that might be needed and overestimated the amount of tracking that is occurring. The focus of the paper was not to establish prevalence of activities but rather explore approaches that adults who are legally blind could use to track weight management–related behaviors.

### Future Directions

As noted earlier, part of this sample was technologically savvy and active in pursuing healthy lifestyle habits. It was clear from this select group that adults with low vision can be creative, resourceful, and proactive in addressing lifestyle-based health behaviors. Results from this study should inform further efforts to develop tailored, evidence-based weight loss program for those with low vision. This would include ensuring that features in wellness programs and apps are accessible to those with low vision and provide the ability to examine data for trends across time as well as share data with health care providers, wellness coaches, or rehabilitation specialists. Weight loss programs and smartphone apps should provide adults who are legally blind with an equivalent user experience compared to those who do not have vision impairments.

## Abbreviations

MVPA: moderate to vigorous physical activity

## Data Availability

Participants granted permission for data to be reported in the aggregate by the study team. None of the participants granted permission for data sharing.
